# Bidirectional glenn surgery without palliative pulmonary artery banding in univentricular heart with unrestricted pulmonary flow. Retrospective multicenter experience

**DOI:** 10.1186/s13019-024-02572-7

**Published:** 2024-02-06

**Authors:** Gaser A. Abdelmohsen, Hala A. Gabel, Rawan M. Alamri, Ahmed Baamer, Osman O. Al-Radi, Aliaa Binyamin, Ahmed A. Jamjoom, Ahmed F. Elmahrouk, Saud A. Bahaidarah, Naif A. Alkhushi, Mohamed H. Abdelsalam, Hossam Ibrahim, Ahmed R. Elakaby, Adeep Khawaji, Abdullah Alghobaishi, Khadijah A. Maghrabi, Zaher F. Zaher, Jameel A. Al-Ata, Ahmad S. Azhar, Ahmed M. Dohain

**Affiliations:** 1 Pediatric Cardiology Division, Department of Pediatrics, King Abdulaziz University, P.O.BOX: 80215, Jeddah, 21589 Saudi Arabia; 2 Pediatric Cardiology Division, Department of Pediatrics, Kasr Al Ainy School of Medicine, Cairo University, 99 El-Manial St., Cairo, 11451 Egypt; 3 Cardiac Surgery Division, Department of Surgery, King Abdulaziz University, P.O.BOX: 80215, Jeddah, 21589 Saudi Arabia; 4 Division of Cardiac Surgery, Cardiovascular Department, King Faisal Specialist Hospital and Research Center, P.O. Box 40047, Jeddah, 21499 Saudi Arabia; 5 Cardiothoracic Surgery Department, Tanta University, Tanta, Egypt; 6 Cardiology Department, Benha University, Benha, Egypt; 7 Pediatric department, Al-Azhar University, Cairo, Egypt; 8 Department of Pediatrics, King Fahad Armed Forces Hospital, Jeddah, Saudi Arabia

**Keywords:** Single ventricle, Unrestrictive pulmonary flow, Pulmonary artery banding, Bidirectional Glenn

## Abstract

**Background:**

Although pulmonary artery banding (PAB) has been generally acknowledged as an initial palliative treatment for patients having single ventricle (SV) physiology and unrestrictive pulmonary blood flow (UPBF), it may result in unfavorable outcomes. Performing bidirectional Glenn (BDG) surgery without initial PAB in some selected cases may avoid the complications associated with PAB and reduce the number of operative procedures for these patients. This research aimed to assess the outcome of BDG surgery performed directly without doing initial PAB in patients with SV-UPBF.

**Methods:**

This Multicenter retrospective cohort includes all patients with SV-UPBF who had BDG surgery. Patients were separated into two groups. Patients in Group 1 included patients who survived till they received BDG (20 Patients) after initial PAB (28 patients), whereas patients in Group 2 got direct BDG surgery without first performing PAB (16 patients). Cardiac catheterization was done for all patients before BDG surgery. Patients with indexed pulmonary vascular resistance (PVRi) ≥ 5 WU.m^2^ at baseline or > 3 WU.m^2^ after vasoreactivity testing were excluded.

**Results:**

Compared with patients who had direct BDG surgery, PAB patients had a higher cumulative mortality rate (32% vs. 0%, *P* = 0.016), with eight deaths after PAB and one mortality after BDG. There were no statistically significant differences between the patient groups who underwent BDG surgery regarding pulmonary vascular resistance, pulmonary artery pressure, postoperative usage of sildenafil or nitric oxide, intensive care unit stay, or hospital stay after BDG surgery. However, the cumulative durations in the intensive care unit (ICU) and hospital were more prolonged in patients with BDG after PAB (*P* = 0.003, *P* = 0.001respectively).

**Conclusion:**

Direct BDG surgery without the first PAB is related to improved survival and shorter hospital stays in some selected SV-UPBF patients.

## Highlight

**Key findings:**

In addition to the advantage of fewer thoracotomies and their adverse consequences, direct BDG surgery without initial PAB is linked with improved survival and a cumulative shorter hospital stay in some selected cases.

**What is known, and what is new?:**

Although pulmonary artery banding (PAB) is generally accepted as an initial palliative treatment for patients with single ventricle (SV) physiology and unrestricted pulmonary blood flow (UPBF), it may have unfavorable outcomes. Bidirectional Glenn (BDG) surgery performed without initial PAB may prevent complications associated with PAB and reduce the number of surgical procedures required for these patients.

**What is the implication, and what should change now?:**

Some patients with SV-UPBF may go directly to BDG surgery without initial PAB if the hemodynamic measurements by cardiac catheterization are favorable.

## Introduction

Patients with univentricular heart and unrestrictive pulmonary blood flow (UPBF) usually develop heart failure symptoms after the physiological decrease of pulmonary vascular resistance that occurs after birth [1, 2]. Pulmonary vascular resistance (PVR) gradually decreases after birth and reaches its lowest value during the early few months of life. With each decrease in PVR, pulmonary blood flow (PBF) increases, causing various symptoms and signs of heart failure. Before bidirectional Glenn (BDG) surgery or partial cavo-pulmonary connection (PCPC) surgery, first-stage palliation aimed to accomplish an unobstructed systemic blood flow with an unobstructed but controlled PBF [3, 4]. In patients with univentricular heart with increased PBF, pulmonary artery banding (PAB) has a significant role in protecting the pulmonary vascular bed from increased pulmonary blood flow, alleviating the heart failure symptoms, and keeping PVR low for the suitability of BDG and Fontan palliation [3–5]. Some institutes reported discouraging short- and long-term results in patients with univentricular hearts palliated with PAB. Some reports even recommended using an aortopulmonary shunt plus surgical interruption of the main pulmonary artery as a first-stage palliative approach, which would offer a more reliable source of PBF and the protection of the pulmonary vascular bed from overflow [6–8]. In patients with aorta arising from the rudimentary ventricle, PAB can result in progressive ventricular hypertrophy, causing more restriction of the interventricular communication with time; these patients may require Damus Kay Stansel (DKS)operation or enlargement of the ventricular septal defect (VSD) at the time of BDG [3, 4, 6, 9–11]. BDG surgery should not be done until the PVR reaches its lowest level. In our institute, some patients with univentricular hearts and increased pulmonary blood flow went directly to BDG (when PVR was expected to be low) without initial PAB. This study aimed to evaluate the outcome of patients with univentricular hearts with increased pulmonary flow who underwent BDG as the first palliative surgery without previous PAB in 2 pediatric cardiac centers.

## Methods

### Patients’ selection and data collection

Informed consent was taken prospectively from the patients’ legal guardians on hospital admission, and the local ethical committee approved the study. From January 2012 to January 2023, all patients with SV-UPBF who underwent BDG surgery were included in this retrospective case-control cohort. Patients exhibiting signs of heart failure, such as shortness of breath, especially while feeding, interrupted feeding, diaphoresis during feeding, lung plethora on chest x-ray, and non-obstructed pulmonary flow on echocardiography, were considered unrestricted pulmonary flow. Patients were then divided into two groups. Patients in Group 1 included patients who survived till they received BDG (20 Patients) after initial PAB (28 patients). In contrast, patients in Group 2 included patients who got direct BDG surgery without performing PAB (16 patients). Cardiac catheterization was done for all patients before BDG surgery. Patients with PVRi ≥ 5 WU.m^2^ at baseline or > 3 WU.m^2^ after vasoreactivity testing were excluded. Data were collected from the patients’ medical records; the data included clinical data like age, weight, diagnosis, surgical data, and postoperative data like duration of intensive care unit stay, hospital stay, cumulative duration of ICU and hospital stay (Stay period after PAB + duration of stay after BDG), mechanical ventilation duration, complications, and survival.

#### Surgical technique for BDG surgery

A midline sternotomy was done, and the thymus was excised. The pericardium was opened, and the aorta, superior vena cava (SVC), and inferior vena cava (IVC) were cannulated. Cardiopulmonary bypass (CPB) was started. The aorta was clamped, and cardioplegia was given. In cases requiring atrial septectomy like tricuspid valve atresia, the right atrium was opened, the atrial septum was excised then the right atrium was closed. The SVC was then divided, and the azygous vein was doubly clipped. The superior aspect of the right pulmonary artery (RPA) was incised; the SVC was divided and anastomosed to the superior aspect of the RPA. The anterior half of the anastomosis was done with interrupted stitches. In cases of BDG, after PAB, the main pulmonary artery was divided, the proximal end was oversewn, the distal end was incised into both pulmonary artery branches, and the autologous pericardium was used to augment the pulmonary artery branches. In cases needing DKS as an additional procedure to the BDG, the aorta was cannulated high, and both cavas were cannulated. The main pulmonary artery was amputated, and the distal stump was closed. The aorta was incised, and a U-shaped flap was created to accommodate the DKS connection with the main pulmonary artery proximal stump. CPB was weaned off. Protamine was given. Two chest drains and pacing wires were inserted, and the chest was closed.

### Statistical analysis

The statistical analysis was performed using IBM SPSS Statistics for Windows, version 26.0 (IBM Corp., Armonk, NY, USA) and Jamovi software (2021). (Version 2.2) [Computer Software] jamovi. Retrieved from https://www.jamovi.org. Using the Shapiro-Wilk test, we assessed the normality of the numerical variables. We presented our non-normally distributed numerical data as a median and interquartile range (25th percentile, 75th percentile). Variables of nominal type were expressed as numbers or numbers and percentages. We analyzed the group comparisons using the non-parametric Mann-Whitney U test for numeric variables and the Chi-square and Fisher exact tests for categorical variables. Survival was assessed using the Kaplan–Meier survival analysis. The *P* value was considered statistically significant if it was less than 0.05.

## Results

Twenty-eight patients with SV-UPBF had PAB as a preliminary palliative PAB procedure before BDG surgery. Eight of these 28 individuals (8/28) died after PAB. The remaining 20 patients who lived until BDG were compared with 16 patients with SV-UPBF who received direct BDG without initial PAB.

As demonstrated in Table 1, patients who underwent direct Glenn surgery without PAB were younger (median age four months) and had a higher QP/QS ratio than those who underwent BDG after initial PAB. There were no statistically significant differences between patient groups regarding gender, anatomical diagnosis, dominant ventricle, degree of atrioventricular valve insufficiency, oxygen saturation before BDG surgery, or hemodynamic parameters measured during cardiac catheterization and before BDG surgery (Table 1).

**Table 1:** Demographic and preoperative data of patients’ groups at Glenn surgery

	Group 1 Glenn-PAB (*n* = 20)	Group 2 Glenn-No PAB (*n* = 16)	*P* value
**Age (months)**	17.3(9.4–23)	4(2.1–5.2)	0.0001*
**Weight (Kg)**	8(6.6–9.3)	4.6(4-6.2)	0.001*
**Male/female, n (%)**	10(50)/10(50)	10(69)/6(31)	0.324
**Diagnosis, n (%)**			0.342
DORV-remote VSD/hypoplastic ventricle	2(10)	3(18.8)	
Tricuspid atresia	3(15)	6(37.5)	
DILV-TGA	2(10)	2(12.5	
Unbalanced AVSD	3(15)	1(6.3)	
Mitral atresia	3(15)		
other	7(35)	4(25)	
**Dominant ventricle, n (%)**			0.552
LV	13(65)	13(81.3)	
RV	5(25)	2(12.5)	
Indeterminate	2(10)	1(6.3)	
**AV valve regurgitation, n (%)**			0.439
Non-trivial	10(50)	10(62.6)	
Mild	3(15)	4(25)	
Moderate	6(30)	2(12.5)	
Severe	1(5)		
**Pre-Glenn oxygen saturation**	85(81–89)	85(76–90)	0.798
**Pre-Glenn catheterization data**			
PVRi (WU.m^2^) on room air	2.3(1.3–2.8)	1.3(1–4)	0.535
QP/QS	1.10(0.7–1.7)	3.7(3-4.7)	0.0001*
Mean PAP (mmHg)	20(16–25)	25(24–36)	0.089
Dominant ventricle EDP (mmHg)	12(7–15)	10(7–12)	0.285

***Abbreviations***: AV: atrioventricular, AVSD: atrioventricular septal defect, DILV: double inlet left ventricle, DORV: double outlet right ventricle, EDP: end-diastolic pressure, LV: left ventricle, PAP: pulmonary artery pressure, PVRi: indexed pulmonary vascular resistance, QP/QS: pulmonary to systemic flow ratio, RV: right ventricle, TGA: transposition of great arteries, VSD: ventricular septal defect, *: statistically significant

Patients with Glenn-PAB underwent their previous PAB at a median age of 1.5 months (minimum 15 days, maximum seven months); this group had CPB and aortic cross-clamping time during BDG longer than patients who underwent BDG surgery without initial PAB (*P* < 0.05). Furthermore, Pulmonary artery plasty as an adjunctive intervention during BDG surgery was higher in group 1 (10 out of 20 cases). In contrast, atrial septectomy was more prevalent during BDG surgery in group 2 (12 out of 16 cases), as shown in Table 2.

**Table 2:** Operative and postoperative data of patient groups following Glenn surgery

	Group 1 Glenn-PAB (*n* = 20)	Group 2 Glenn-No PAB (*n* = 16)	*P* value
**CPB time, minutes**	72(64–92)	50(35–68)	0.018*
**Aortic cross-clamping time, minutes**	47(29–60)	22(8.5–39)	0.012*
**Type of Glenn, (%)**			0.529
Right BDG	15(75)	14(87.5)	
Left BDG	1(5)		
Bilateral Glenn	4(20)	2(12.5)	
**Additional procedures, n**			
PA plasty	10	3	
Atrial septectomy	6	12	
AVV repair	1	-	
DKS	9	3	
PAB tightening	1	-	
PA amputation	10	13	
PPM	1		
**Early redo surgery, n (%)**			0.413
Glenn revision	1(5)	-	
ECMO	1(5)	-	
**Postoperative data after Glenn**			
Delayed chest closure, n (%)	3(15)	2(12.5)	0.829
iNO, n (%)	3(15)	5(31)	0.422
Sildenafil use, n (%)	4(20)	6(37.5)	0.285
Postoperative CVP (mean PAP), mmHg	17(15–23)	18(20–23)	0.582
Duration of pleural drainage, days	5(3.5–10)	9(6–12)	0.080
Duration of mechanical ventilation, Days	9(3–22)	11(4–23)	0.780
Postoperative phrenic palsy, n (%)	-	1(5%)	
Failed extubation, n (%)	1(5)	-	
Chylothorax, n (%)	3(15)	2(12.5)	0.829

***Abbreviations***: AVV atrioventricular valve, BDG: bidirectional Glenn, CPB: cardiopulmonary bypass, CVP: central venous pressure, DKS: Damus Kay Stansel, ECMO: extracorporeal membrane oxygenation, iNO: inhaled nitric oxide, PA: pulmonary artery, PAB: pulmonary artery band, PPM permanent pacemaker, *: statistically significant

**Fig. 1:**
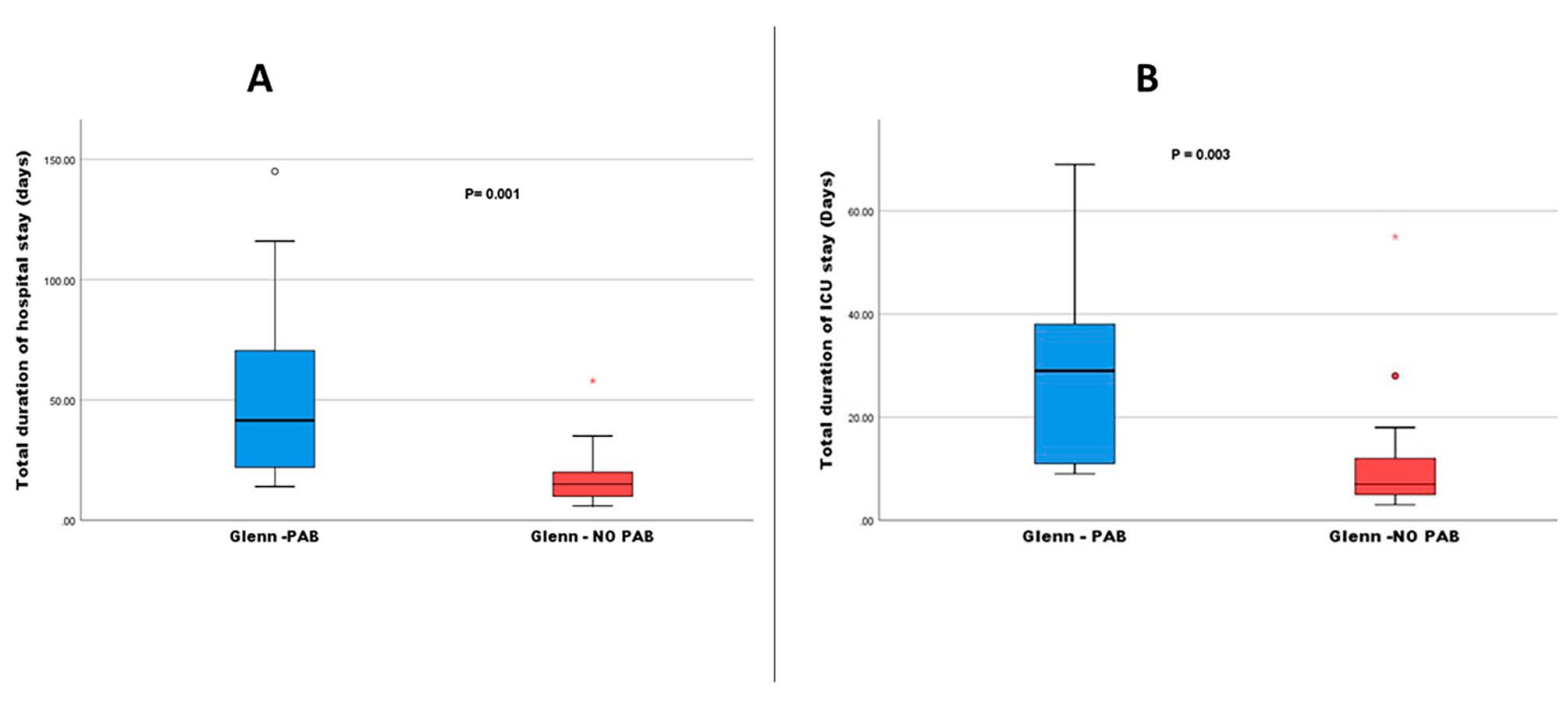
The cumulative length of hospital stay (**A**) and ICU stay (**B**) of patients groups The cumulative duration of ICU and hospital stays was longer for PAB-Glenn patients, as demonstrated in Fig. 1. PAB: Pulmonary artery banding; ICU: intensive care unit

Regarding the postoperative period after BDG surgery, there were no significant differences between the patient groups in terms of the frequency of inhaled nitric oxide administration, the use of sildenafil, postoperative Glenn pressure (measured through an internal jugular veinous catheter), the duration of mechanical ventilation, the duration  of postoperative pleural drainage or the frequency of postoperative complications (Table 2). Although the hospital and ICU admission durations after BDG surgery were comparable between the two groups, the cumulative durations of ICU and hospital stay in group 1 (PAB, BDG) were significantly longer compared to group 2 (*P* = 0.003, *P* = 0.001 respectively, Tables 2 and 3; Fig. 1).

**Table 3:** Outcome of patients group

	Group 1 Glenn-PAB (*n* = 20)	Group 2 Glenn-No PAB (*n* = 16)	*P* value
Post BDG duration of ICU stay, days	7(5-16.5)	7(5–16)	0.792
Post BDG hospital stay, days	11(9–22)	15(10–20)	0.286
Cumulative duration of ICU length of stay, days	29(11–39)	7(5–16)	0.003*
Cumulative duration of hospital stay, days	35(19–68)	15(10–20)	0.001*
Mortality after BDG, n (%)	1(5)	-	
Fontan completion, n (%)	15(75)	10(62.5)	0.907
	**All PAB** **(** ***n*** **= 28)**	**Glenn-No PAB** **(** ***n*** **= 16)**	*P*-value
Cumulative mortality, n (%)	9(32)	0	0.016*

***Abbreviations***: BDG: bidirectional Glenn, ICU: intensive care unit, PAB: pulmonary artery band, *: statistically significant

**Fig. 2:**
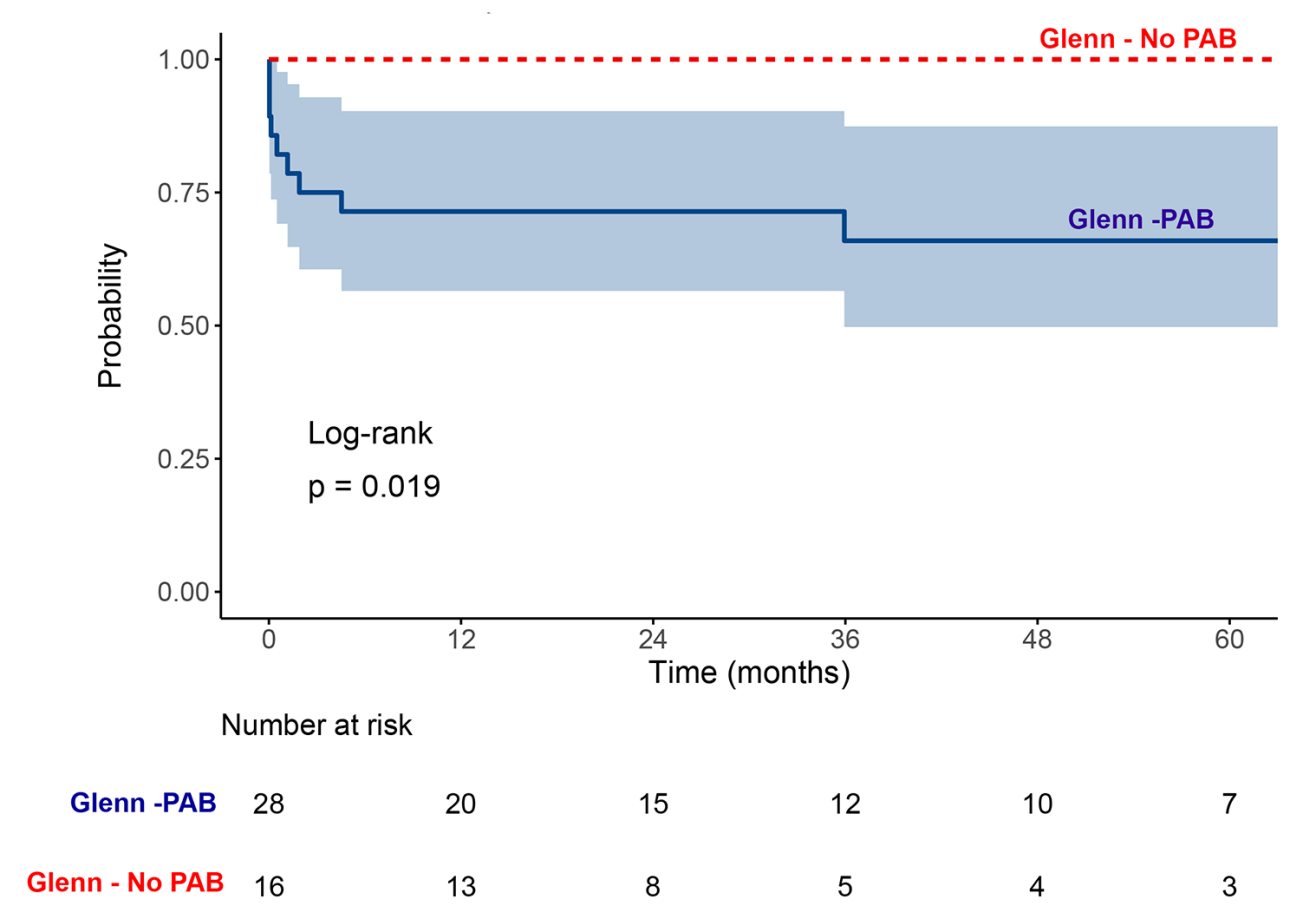
Kaplan Meier survival analysis of patients’ groups As demonstrated, mortality is high among Glenn-PAB patients, particularly after PAB. PAB: pulmonary artery banding

Although there was no significant difference between the groups in terms of mortality after BDG, of the 28 patients who underwent PAB, eight patients (28.5%) died after PAB and before BDG surgery, and one (3.5%) died after BDG, resulting in a cumulative mortality rate of 32% compared to no mortality in patients who underwent BDG surgery without initial PAB (*P* = 0.016) as shown in Table 3. The cause of death after PAB was intracranial hemorrhage in one patient, low cardiac output in 3 patients (one of them required extracorporeal membrane oxygenation /ECMO support), prolonged hospital stays, sepsis, and disseminated intravascular coagulopathy in 3 patients, and infective endocarditis in one patient. Figure 2 illustrates Kaplan Meier survival analysis showing higher mortality after PAB. In group 1, 15 patients (75%) underwent Fontan surgery, while in group 2, 10 (62.5) underwent Fontan surgery. No mortality was reported after the Fontan shunt.

## Discussion

### The traditional PAB in patients with SV-UPBF, do we still need it?

PAB has been considered a universally accepted strategy for univentricular palliation in patients with SV-UPBF. It can keep pulmonary vascular resistance and pulmonary artery pressure at appropriate levels for patients with SV-UPBF and is planned for future Fontan surgery. On the other hand, PAB has many drawbacks, including the inability to adequately protect the pulmonary circulation, pulmonary arterial distortion, and pulmonary valve degeneration and regurgitation. PAB may also promote ventricular hypertrophy, which may accelerate the restriction of interventricular communication, causing early outflow tract obstruction of the subaortic ventricle as in cases with double inlet left ventricle (DILV) or tricuspid atresia and TGA [3–6, 9].

Numerous centers have documented unfavorable outcomes in individuals with a single ventricle following PAB. Theoretically, utilizing BDG as the initial palliative treatment for these patients, as opposed to PAB, could avoid the complications of PAB and reduce the number of operative procedures. However, the possibly elevated pulmonary vascular resistance, pulmonary artery pressure, and plethoric lungs secondary to UPBF may be reasons for concern.

### PVR and the age at which direct BDG surgery is optimal

As reported in the literature, a low PVRi (≤ 3 WU.m2) is one of the characteristics linked with favorable outcomes following BDG surgery [2, 11]. In this cohort, 11/16 patients in group 2 got direct BDG without initial PAB between the ages of 2 to 4 months. As the PVR is likely at its minimal value at this age, this age may be optimal for performing direct BDG. It may be risky to perform direct BDG after six months or before two months of life because the PVRi may exceed the safe values (≤ 3 WU.m2) [2, 11, 12]. In this cohort, 3/16 patients in group 2 underwent direct BDG at 10–11 months and three patients before the age of 2 months; fortunately, all these patients had a PVRi ≤ 5 WU.m2 on room air and < 3 following vasoreactivity testing.

### BDG surgery and additional surgical procedures

Other surgical procedures may be necessary during BDG surgery, such as atrial septectomy and pulmonary artery plasty. In this cohort, group 1 patients had longer CPB and aortic cross-clamping times than other patients; this may have been due to adhesions associated with previous PAB surgery and the need for pulmonary artery plasty, which was frequently required due to pulmonary arterial distortion developed after PAB (Table 2).

PAB may accelerate the constriction of interventricular communication, resulting in early obstruction of the subaortic ventricle’s outflow tract; this may explain why 9/20 patients (45%) in group 1 underwent an extra Damus Kay Stansel procedure compared to 3/16 patients (18.7%) in group 2 [3, 5].

### The postoperative period after BDG surgery

Regarding the Glenn pressure measured by the central venous catheter and the use of pulmonary vasodilators after BDG, the postoperative course of both groups was comparable; this may reflect the inadequacy of PAB to adequately protect pulmonary circulation in some patients in group 1. Although there was no statistically significant difference between the groups, 37.5% of patients in Group 2 were given sildenafil, and 31% were given iNO therapy. This might be due to poor lung compliance in patients with high pulmonary flow who do not have prior pulmonary artery banding, causing a relative increase of PVR, particularly following cardiopulmonary bypass in the early postoperative period. Furthermore, some intensivists utilize iNO following Glenn procedures if the mean PAP is high and there is postoperative desaturation to enhance flow across the Glenn and, therefore, saturation. The administration of pulmonary vasodilators lacked a standardized protocol and was primarily determined subjectively on a case-by-case basis. The clinical course was taken into consideration when making this decision. However, in general, pulmonary vasodilators were administered to patients who exhibited significant desaturation following BDG, had high mean pulmonary artery pressure as measured through a central line inserted into the superior vena cava, experienced high pleural drainage, or demonstrated elevated pulmonary artery pressure (> 15 mmHg) or pulmonary vascular resistance (> 3 WU.m^2^) on room air during pre-Glenn catheterization plus postoperative desaturation. The absence of statistically significant differences between patient groups in terms of ICU length of stay, hospital length of stay, duration of mechanical ventilation, and postoperative consequences could cast some doubt on the efficacy of PAB in protecting pulmonary circulation in some patients.

### The outcome of direct BDG vs. PAB followed by BDG surgery

**Fig. 3:**
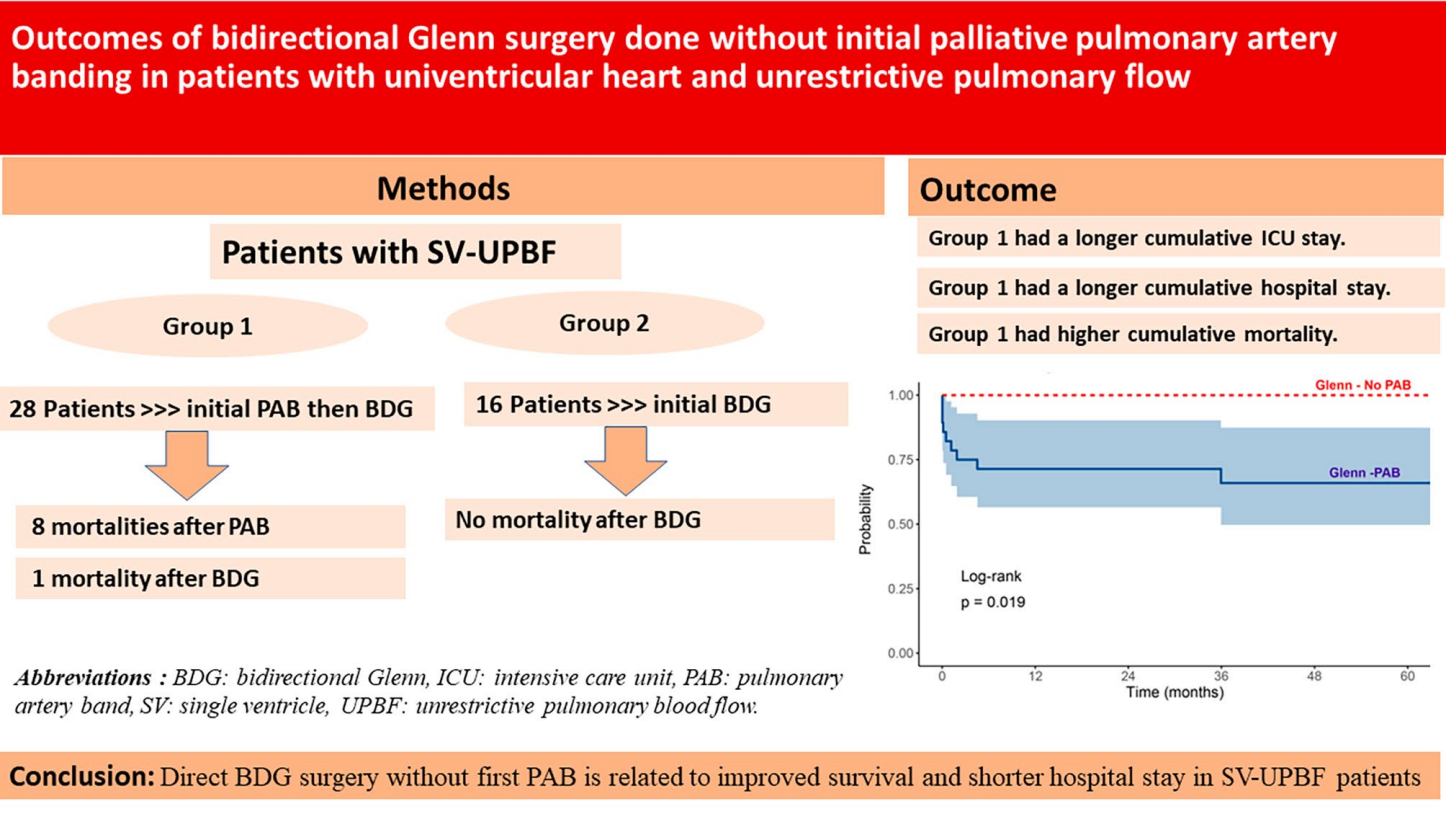
Graphical abstract summarizes the current study

In this study, the cumulative mortality was higher after PAB, and the cumulative length of hospitalization after PAB followed by BDG was significantly longer. Applying PAB can increase the afterload on the ventricle that already had a high preload due to increased pulmonary blood flow and venous return. This increase in afterload after PAB can lead to ventricular hypertrophy and geometrical ventricular changes with an abnormal volume-to-mass ratio, causing ventricular dysfunction. This may explain the unfavorable outcome after PAB [13, 14]. These findings should prompt us to reconsider the necessity of PAB as an initial palliative measure for patients with SV-UPBF. Application of Direct BDG without performing PAB in the presence of appropriate PVR evaluated by cardiac catheterization in some selected cases may be superior to initial PAB followed by BDG because it results in fewer procedures, shorter hospital stays, lower cost, and less psychosocial frustration for patients and their families with each thoracotomy. Figure 3 shows a graphical abstract that summarizes the study.

### Strength and limitations

This study presented a multicenter experience with a novel approach to managing patients with SV-UPBF; no previous studies had addressed this topic. The primary limitations of this cohort are its retrospective design, so bias could not be avoided, the small number of patients recruited, and the absence of patients with SV-UPBF and high PVR. The study was also limited by a lack of who needed PAB but did not undergo it and died before BDG. For an accurate evaluation of the efficacy of both surgical treatments available to patients with SV-UPBF, it is suggested that future randomized controlled trials be conducted.

## Conclusions

Performing direct BDG surgery without initial PAB in certain patients with SV-UPBF, particularly those with low PVR, may potentially yield improved survival rates, reduced cumulative ICU stays, and shorter hospital stays compared to the approach of performing PAB followed by BDG surgery.

## Data Availability

The data supporting the findings of this research are accessible upon reasonable request from the corresponding author if patients’ data privacy is not compromised.

## Abbreviations

AV: Atrioventricular
AVSD: Atrioventricular septal defect
AVV: Atrioventricular valve
BDG: Bidirectional Glenn
CPB: Cardiopulmonary bypass
CVP: Central venous pressure
DILV: Double inlet left ventricle
DKS: Damus Kay Stansel
DORV: Double outlet right ventricle
ECMO: Extracorporeal membrane oxygenation
EDP: End-diastolic pressure
ICU: Intensive care unit
iNO: Inhaled nitric oxide
IVC: Inferior vena cava
PA: Pulmonary artery
PAB: Pulmonary artery banding
PAP: Pulmonary arterial pressure
PBF: Pulmonary blood flow
PCPC: Partial cavo-pulmonary connection
PPM: Permanent pacemaker
PVR: Pulmonary vascular resistance
PVRi: Indexed pulmonary vascular resistance
QP/QS: Pulmonary to systemic flow ratio
RPA: Right pulmonary artery
SV: Single ventricle
SVC: Superior vena cava
SV-UPBF: Single ventricle with unrestrictive pulmonary blood flow
TGA: Transposition of great arteries
UPBF: Unrestrictive pulmonary blood flow
VSD: Ventricular septal defect
WU: Wood unit
